# Prevalence and incidence of retinopathy in elderly diabetic patients receiving early diagnosis and treatment

**DOI:** 10.3892/etm.2013.1021

**Published:** 2013-03-20

**Authors:** XIN LI, ZHAOYAN WANG

**Affiliations:** 1 Departments of Internal Medicine, Chinese PLA General Hospital, Beijing 100853, P.R. China; 2 Ophthalmology, Chinese PLA General Hospital, Beijing 100853, P.R. China

**Keywords:** diabetic retinopathy, diabetes, elderly, incidence, prevalence

## Abstract

Diabetic retinopathy (DR) is one of the most common and specific complications of diabetes. Thus, intervention is required to lower the incidence and prevalence of sight-threatening retinopathy. The aim of this study was to investigate the prevalence and incidence of diabetic retinopathy in elderly diabetic patients receiving early diagnosis and proper treatment and to explore risk factors for DR. From May 2005 to May 2011, fundus examination was performed for elderly diabetic patients during routine medical examinations. The presence of a microaneurysm or more severe presentations was used to diagnose DR, which was followed by fundus fluorescein angiography. Logistic regression analysis was employed to analyze the risk factors for DR development within 5 years. A total of 2,194 diabetic patients were recruited and followed-up for a mean of 5.8 years. The prevalence of DR was 15.38–16.20% and the incidence of DR was 8.38/1,000 person-years. Logistic multiple stepwise regression revealed that fasting plasma glucose (FPG), mean arterial pressure (MAP), duration of diabetes, body mass index (BMI) and microalbuminuria (MAU) were significantly associated with the occurrence of DR (all P<0.05). In the present study, the prevalence and incidence of DM were higher compared with those reported in the general population; however, the prevalence and incidence of DR were lower compared with those reported in similar studies. This suggests that favorable control of blood glucose, blood pressure and blood lipids effectively prevents the occurrence of DR in diabetic patients.

## Introduction

Diabetic retinopathy (DR) is one of the most common and severe complications of diabetes mellitus (DM) (1). Since the development of type 2 DM is progressive, the impact of DR on vision varies according to the stage of DR. In certain patients, DR is already present at the time of DM diagnosis. If DR is not promptly diagnosed and treated, proliferative diabetic retinopathy (PDR) induces severe visual impairment, resulting in treatment difficulty (2). Since PDR has become a main cause of blindness in adults, an increased focus on the early prevention and control of DR has emerged (3).

It is well established that planned and organized screening and timely treatment of DR among patients with DM, particularly those with a high risk of DR, are effective methods for reducing the population burden of this condition. However, screening and timely treatment is a long-term process for clinicians and patients. To date, there have been no studies on DR that include a large sample size, long-term follow-up and well-controlled risk factors. In response to this evidence gap, the present study recruited elderly patients with DM who received timely diagnosis and strictly controlled treatment at the Chinese PLA General Hospital (Beijing, China). The prevalence and incidence of DR among elderly DM patients receiving early diagnosis and proper treatment was assessed. Due to the study characteristics, the results accurately reflect the actual incidence of DR in elderly patients and may help to reveal the risk factors of DR in diabetic patients.

## Materials and methods

### Patients

A total of 1,804 patients with DM receiving medical examination at the Department of Ophthalmology, Chinese PLA General Hospital in May 2005 were recruited for the present study. Additionally, 309 and 81 patients were recruited in 2006 and 2007, respectively, resulting in a total enrollment at baseline of 2,194 patients. All patients resided in Beijing for >10 years and had a mean age of 72.5 years (range, 60–97 years). Patients with DM were diagnosed at the Department of Endocrinology. Patients underwent annual re-examination and follow-up was terminated in May 2011. Patients recruited in 2005 and 2011 were followed up for 6 and 5 years, respectively. During the follow-up period, 90 patients succumbed to various causes. More than 90% of the remaining patients received five physical examinations during the follow-up period.

### Assessments

The assessments were conducted by experts on fundus diseases and endocrinology. A form was used to record the clinical information of each patient, including personal history (smoking, alcohol consumption and physical activity), family history, history of present illness and information associated with other diseases. Other outcomes assessed and recorded included the duration of DM, medical history of DM, body weight, height, body mass index (BMI), resting blood pressure (BP; measured while seated after 30 min of rest), fasting plasma glucose (FPG), total cholesterol (TC), triglyceride (TG), high-density lipoprotein cholesterol (HDL-C) and microalbuminuria (MAU).

### Detection methods

Patients with endocrinologically-confirmed DM received a fundus examination following mydriasis. The fundus was initially observed under an indirect ophthalmoscope. In the presence of a microaneurysm, fundus fluorescein angiography was performed to determine the degree of DR.

### Diagnostic criteria

DR was staged according to the criteria developed at the Conference on Fundus Diseases (1984), which consist of six stages: three non-proliferative (first three stages) and three proliferative stages (last three stages). However, some findings in the non-proliferative stages may also be present in the proliferative stages. In addition, macular edema was classified into mild, moderate and severe (4,5).

### Statistical analysis

All data are expressed as mean ± standard deviation. Analysis of variance was used to compare continuous data and a *χ*^2^ test was used to compare rates between the different groups. Logistic multiple stepwise regression analysis was performed to analyze the correlation between baseline parameters and DR risk. The incidence of DR was analyzed by employing the person-years method. Time was defined as the interval between baseline and the occurrence of DR. For patients without DR, time was defined as the interval between baseline and the end of the study. Statistical analysis was performed using Stat version 5.0 (SAS Institute Inc., Cary, NC, USA).

## Results

### Baseline characteristics

Data from 1,687 elderly males and 507 elderly females who were retired cadres were used for analysis. Patients frequently participated in activities or were physically active and consumed little to no alcohol or tobacco. BMI, BP, FPG, TC, TG and HDL-C levels were generally favorable among patients, all of whom were continuously treated with aspirin and vitamins (Table I).

### Prevalence of DR

As shown in Table II, among the elderly patients visiting the Department of Endocrinology of our hospital in 2005, the prevalence of DR was 15.96% (range, 15.38–16.20%) over the following 6 years. Although the prevalence increased slightly, this change was not statistically significant (*χ*^2^=5.8422, P=0.9588).

### Incidence of DR

Over a mean follow-up of 5.8 years, 87 new cases of DR were noted out of 1,791 participants who were free of DR at baseline. Thus, the incidence of DR in our study was 8.38/1000 person-years.

### Logistic multiple regression analysis of DR risk factors

As shown in Table III, the clinical information collected at baseline was used to predict the occurrence of DR during the study. DR (yes=1, no=0) and impaired glucose tolerance (IGT; yes=1, no=0) served as dependent variables. Medical history (cardio/cerebrovascular diseases, hypertension and hyperlipidemia; yes=1, no=0), family history of diabetes (yes=1, no=0), duration of DM (≥10 years =1, <10 years =0), age, FPG, post-plasma glucose (PPG), BMI, TC, TG, HDL-C, BP and MAU served as independent variables. Logistic stepwise regression analysis revealed that five risk factors were closely associated with the occurrence of DR, including duration of DM, BMI, BP, FPG and MAU (all P<0.05; Table IV).

## Discussion

In the present study, elderly DM patients in Beijing were recruited and followed-up for 6 years to determine the incidence and prevalence of DR. The prevalence of DR was obtained over 6 consecutive years. Previous epidemiological studies were largely cross-sectional and thus only reflect the prevalence of DR during a specific year (6,7). Previously, the prevalence of DR reported by epidemiological studies has varied, at least partly due to different patient populations and variable sample sizes. In an Italian multi-center epidemiological study, the prevalences of DR and PDR in patients with type 2 DM were reported to be 34.6 and 6.2%, respectively (7). In France, an analysis of ten epidemiological studies of DM reported that the prevalences of DR and PDR were 33.0 and 3.3%, respectively (7). In China, Wu *et al* reported that the prevalences of DR and PDR were 27.8 and 4.2%, respectively (6). In the present study, the prevalences of DR and PDR were 15.38–16.20% and 1.5%, respectively. Thus, the rates of DR and PDR reported in our study are significantly lower than those previously reported (8–11). In 2003, Pan *et al* reported that the prevalence of DM in military retired cadres in Beijing, who were >60 years of age, fluctuated between 17.7 and 28.7% (12). These rates are generally higher than those reported in previous studies in China (13,14). These findings suggest that while the prevalence of DM is relatively high among these elderly patients, the incidences of DR and PDR remain relatively low. A number of factors may explain these observations. First, the subjects in the present study have favorable access to medical resources and have a relatively high quality of life and thus may be a biased representation of the general elderly population. These individuals receive free physical examinations twice each year (15,16). Secondly, once DM is diagnosed, these individuals receive proper and timely treatment, resulting in only mild DM among a number of them (17,18). Thirdly, elderly patients have special characteristics of the eye structure (posterior vitreous detachment), resulting in slower progression of DR than that observed among younger patients (19). Additionally, these patients have good knowledge of DM and have shown high compliance with DM treatment. Thus, blood glucose, BP, blood lipids, body weight and smoking are well-controlled. As a result, the incidence of DR and particularly that of PDR among these patients is significantly lower than that of the general population (20–23). During the six years of follow-up in the present study, none of the patients developed grade V or VI DR. Of the patients in the present study, 21 had a long-term (>40 years) history of DM and four of these patients had newly formed blood vessels in the retina. Even among these patients with a long-term history of DM, the prevalence of PDR was <20%. Of the five patients with macular edema, only mild damage to the posterior pole of the retina was observed. Other patients mainly had mild microaneurysm. These results demonstrate that proper control of blood glucose, BP, blood lipids, obesity and smoking effectively prevents the occurrence and development of DR.

## Figures and Tables

**Table I:** Baseline characteristics of the study groups.

	Diabetic subjects (n=2,194)		
Characteristics	NDR (n=1,830)	NPDR (n=336)	PDR (n=28)
Age (years)	72±9	72±10	72±9
Male/female	1481/349	187/149	19/9
Clinical			
Body mass index (kg/m^2^)	26.1±4.2	25.8±3.3	26.2±3.8
Systolic blood pressure (mmHg)	132±18	130±15	134±15
Diastolic blood pressure (mmHg)	70±11	70±12	72±11
Mean arterial pressure (mmHg)	87±13	86±12	88±14
Duration of diabetes (years)	7±5	11±7	17±8
Smoking (%) non/ex/current	1734/93/3	312/23/1	20/8/0
Alcohol (%) non/social/regular	7/1803/20	2/315/19	0/17/11
Exercise (%) sedentary/moderately/active	0/1811/19	2/308/26	0/23/8
Biochemical			
FPG (mmol/l)	6.4±2.3	6.8±3.1	6.8±2.7
HbA1c (%)	6.4±1.1	6.4±1.2	6.5±1.4
Triglyceride (mmol/l)	2.39±1.3	2.75±1.4	2.78±1.6
Total cholesterol (mmol/l)	6.2±2.8	6.3±2.4	6.4±2.6
LDL-cholesterol (mmol/l)	3.34±1.1	3.67±0.8	3.69±1.2
HDL-cholesterol (mmol/l)	1.07±0.4	1.03±0.5	1.04±0.3
Medications			
>1 aspirin daily (%)	91	98	97
Others (%) digoxin/antihypertensive/			
lipid lowering/HRT or OC (%)	84	92	94

NDR, non-diabetic retinopathy; NPDR, non-proliferative diabetic retinopathy; PDR, proliferative diabetic retinopathy; FPG, fasting plasma glucose; LDL, low-density lipoprotein; HDL, high-density lipoprotein; HRT, hormone replacement therapy; OC, oral contraceptive therapy.

**Table II:** Prevalence of diabetic retinopathy (DR) among elderly patients with diabetes mellitus.

Year	Examinees (n)	DR prevalence % (n)	PDR prevalence % (n)
2005	1804	15.96 (288)	1.27 (23)
2006	2103	15.64 (329)	1.24 (26)
2007	2184	15.38 (336)	1.28 (28)
2008	2134	15.65 (334)	1.31 (28)
2009	2077	16.03 (333)	1.49 (31)
2010	2075	16.10 (334)	1.49 (31)
2011	2068	16.20 (335)	1.55 (32)

PDR, proliferative diabetic retinopathy.

**Table III:** Findings in the fundus of elderly patients with diabetic retinopathy (DR; 342 patients, 684 eyes).

Lesions	No. of affected eyes	%
Microaneurysm	425	62.13
Small bleeding spots	284	41.52
Hard exudate	221	32.31
Cotton wool spot	132	19.30
Macular edema	97	14.18
Moderate bleeding spot	32	4.68
Retinal neovascularization	34	4.97
Epiretinal membrane	5	0.73
Neovascularization of the optic disc	1	0.15
Retinal vascular sheath	1	0.15

**Table IV:** Multiple logistic regression analysis of newly diagnosed diabetic retinopathy (DR).

Variable	Partial regression coefficient	Standard error	Odds ratio	P-value
Blood pressure	0.68	0.590	0.42	0.0061
Duration of DM	0.550	0.413	1.40	0.0410
BMI	0.071	0.028	1.20	0.0001
FPG	0.512	0.098	1.58	0.0045
MAU	0.014	0.006	1.06	0.0470

DM, diabetes mellitus; BMI, body mass index; FPG, fasting plasma glucose; MAU, microalbuminuria.
